# Omental Torsion: An Unusual Cause of Right Iliac Fossa Pain and Role of Laparoscopic Management

**DOI:** 10.4021/gr299e

**Published:** 2014-01-15

**Authors:** Ali Tasleem, Qamar Zaman, Daniel A Thomas, John G Payne, Rajab Kerwat, Aftab A Khan

**Affiliations:** a Department of Surgery, Queen Mary Hospital, Sidcup, Kent DA14, UK

**Keywords:** Omental torsion, Acute abdomen, Laparoscopy

## Abstract

Omental torsion is a rare cause of acute abdomen. It usually presents with acute onset right-sided abdominal pain. Adult male between 40 and 50 years of age and obesity are the most common risk factor amongst others. Clinical diagnosis is challenging and difficult to differentiate from more common clinical pathologies such as acute appendicitis and/or acute cholecystitis. Transabdominal imagings such as ultrasonography and/or computed tomography are useful showing typical whirl pattern. Advocated management is surgical excision of torted omentum. Herein, we report a case of primary omental torsion in an adult and a review of current literature. The diagnosis was incidental when patient was undertaken for laparoscopic appendectomy. Only the distal edge of right omentum was torted making a fatty mass of 4 × 3 cm lying on the ascending colon that could have been easily missed if open appendectomy was opted. This case not only highlights the importance of considering torted omentum in differential diagnosis of right-sided abdominal pains but also backs the changing practice to laparoscopic approach for management of right iliac fossa pain.

## Introduction

Omental torsion is a rare cause of acute abdominal pains. Increased awareness of its existence is growing amongst surgeons with changing trends in management of acute abdomen by frequent use of sophisticated transabdominal imaging and laparoscopy. The condition is more common in adults (40 - 50 years) with males twice more likely than females to be affected. Obesity is a common risk factor in all reported cases. Sudden onset right-sided abdominal pain is typical presenting complaint and therefore clinical differentiation from more common right-sided pathologies such as acute appendicitis and acute cholecystitis is very difficult. This report describes an interesting case of omental torsion in an adult patient, where only a small distal edge of right greater omentum was torted. Diagnosis was made incidentally when patient underwent laparoscopic appendectomy.

## Case Report

**Figure 1:**
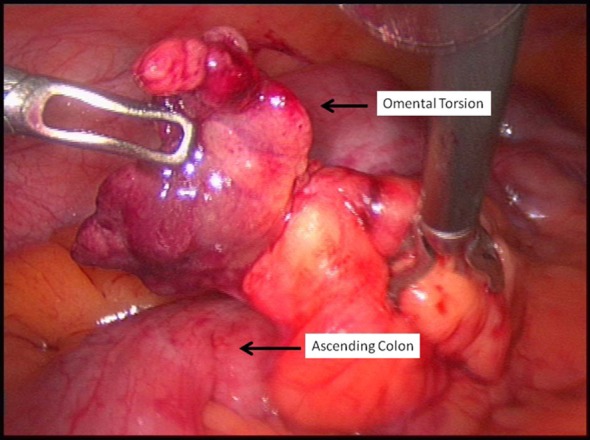
Original location of the torted omental segment. It was located on the ascending colon distal to the appendix.

**Figure 2:**
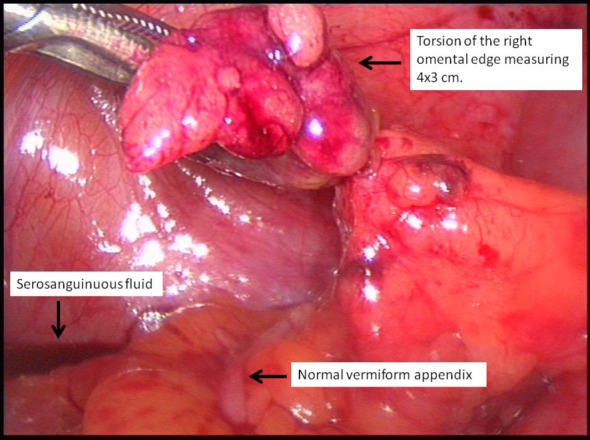
Torted right omental edge and typical serosanguinous fluid. Also note normal looking appendix.

**Figure 3:**
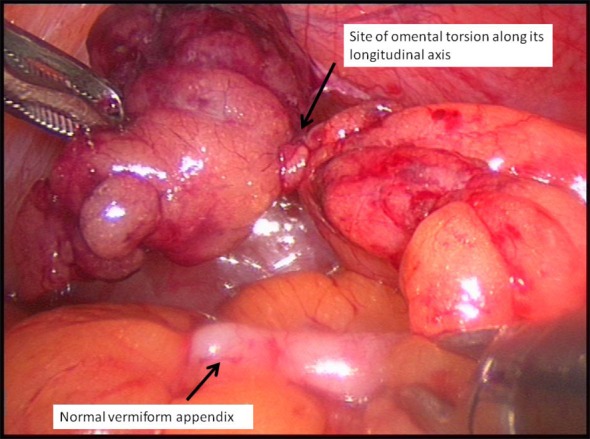
Site of omental torsion.

A 45-year-old gentleman presented with acute onset right iliac fossa pain of 36 h duration. He described the pain as “twisting” in character, non-radiating, constantly present and worsening in severity (verbal score 7/10) not alleviated by simple analgesia. He also complained of nausea and loss of appetite but no new change in bowel habits. He was under investigation for chronic diarrhoea of 10 years. His other relevant medical history included type II diabetes mellitus and hypertension. On examination, the patient was noted to have a large body habitus (BMI 28) and stable observation. Per abdomen there was tenderness, guarding and percussion tenderness in the right iliac fossa. There was no palpable visceromegaly. The urinalysis was negative for any abnormality. All his bloods tests including C-reactive protein and amylase were normal. Based on the clinical findings of localized right iliac peritonism, a provisional diagnosis of acute appendicitis was made and patient was taken for surgery. With changing trends in approach to acute surgery and increased use of laparoscopic intervention in our department, a laparoscopic appendectomy was planned. This revealed a macroscopically normal vermiform appendix with bulky mesoappendix and minimal serosanguinous in pelvis. This prompted examination of other viscera which revealed a torted section of right distal omentum lying 10 cm distal to the appendix between lateral ascending colon and adjacent anterior abdominal wall (Fig. 1), forming a congested, dusky, fatty mass measuring 4 × 3 cm (Fig. 2, 3). There were inflammatory adhesions between the torted omental mass and anterior abdominal wall. Appendectomy and local resection of torted omental segment were carried out. Both specimens were retrieved in a bag through 10 mm umbilical port. Postoperatively, the patient made a good overall recovery apart from superficial wound infection at the umbilical port site which was successfully treated with oral antibiotics. He was discharged in couple of days and has been well on review after 3 months.

## Discussion

Right iliac fossa pain is a common cause of acute surgical admissions. It remains the commonest clinical dilemma for general surgeon and yet there are no standard management strategies. Open appendectomy has been used liberally in the past. However, as evidence gathers, conservative management such as use of scoring systems [1] and imaging techniques [2] has shown benefits. Computed tomography (CT) has shown to decrease negative appendectomies [3]. In cases, where surgical intervention is chosen, laparoscopy is now proven to be far superior to open technique [4]. Diagnostic laparoscopy decreases rates of negative appendectomy [5] and has subsequently increased recognition and reporting of other obscure pathologies like omental torsion.

Omental torsion along its long axis causing vascular compromise is a rare cause of acute abdomen. It was first described by Eittel in 1899 [6] and has been recognized as either primary, which occurs in the absence of associated pathology or more commonly secondary, which is associated with intra-abdominal pathology such as adhesions, omental cyst, hernia or tumors. Our patient represents a case of primary omental torsion. Primary omental torsion has been reported being more common in adults, with peak age between 40 and 50 years, two-thirds of whom are male [7]. It is rare in children and is only found in 0.1 - 0.5% of children undergoing operation for presumptive appendicitis [8]. Obesity is the most common risk factor identified. There are also a number of predisposing factors to primary omental torsion which include anatomic variation of the omentum such as tongue-like omental projections, bifid or accessory omentum and vascular anomalies like redundant omental veins, sudden change in body position, overexertion, overeating or abdominal trauma. Our patient was noted to be obese but none of the other risk factors were identified. Reported literature also shows that most cases present with sudden onset right-sided abdominal pain with localized tenderness and/or guarding mimicking acute appendicitis. This corresponds to common involvement of right side of omentum which is larger, longer and more mobile than left side, making it prone to torsion [9]. Features such as nausea, vomiting, fever and leucocytosis are unusual [10]. However, our patient complained of nausea and loss of appetite which may have been due to pain. Traditionally, the diagnosis of omental torsion was made intraoperatively; however, with changing trends in management of acute abdomen by frequent use of abdominal CT scanning, preoperative diagnosis of omental torsion has increased significantly. A typical CT finding includes focal mass of fat density showing streaks in a whirling pattern, which may be associated with little thickening of adjacent bowel wall and presence of serosanguinous fluid [11]. A similar whirling pattern may be seen in small bowel volvulus but it is usually associated with small bowel obstruction and is centrally located in the mesentery [12]. Traditional management has been localized resection of torted segment of omentum found intraoperatively. The usual operative findings include presence of serosanguinous fluid which should prompt the operator to look for omental torsion [13]. More recently, successful outcomes of conservative management have also been reported [14, 15], but it is dependent on unambiguous radiologic diagnosis [16].

As expected with omental torsions, clinical presentation of our patient was hard to distinguish from acute appendicitis and therefore we opted directly for surgical intervention. Note that our patient complained of nausea and loss of appetite which is not typical of omental torsion. Laparoscopy proved exceptionally useful in detecting torted omental edge. We believe, this diagnosis could have been missed if classical open appendectomy was performed due to the location of torted omental edge and absence of usual operative stigmata.

In conclusion, we present a unique case of omental torsion where the patient presented with right-sided abdominal pain associated with nausea and loss of appetite. Laparoscopy was particularly useful in diagnosis as the torted omental edge was present with minimal amount of typical serosanguinous fluid. Therefore, in view of our experience and current literature, we recommend that a diagnosis of omental torsion should be suspected in patients with right abdominal pain who are fat, 40 and of male gender. CT imaging may help diagnosis by showing typical whirl pattern and presence of serosanguinous fluid. We also recommend preference of laparoscopy as the first surgical intervention for managing right iliac fossa pains. Surgical resection of affected omental segment has shown good results.
